# High Resolution Melting (HRM) for High-Throughput Genotyping—Limitations and Caveats in Practical Case Studies

**DOI:** 10.3390/ijms18112316

**Published:** 2017-11-03

**Authors:** Marcin Słomka, Marta Sobalska-Kwapis, Monika Wachulec, Grzegorz Bartosz, Dominik Strapagiel

**Affiliations:** 1 Biobank Lab, Department of Molecular Biophysics, Faculty of Biology and Environmental Protection, University of Łódź, Pilarskiego 14/16, 90-231 Łódź, Poland; marcin.slomka@biol.uni.lodz.pl (M.S.); marta.sobalska@biol.uni.lodz.pl (M.S.-K.); 2 BBMRI.pl Consortium, 54-066 Wrocław, Poland; 3 Department of Molecular Biophysics, Faculty of Biology and Environmental Protection, University of Łódź, Pomorska 141/143, 90-236 Łódź, Poland; monika.caban@biol.uni.lodz.pl (M.W.); gbartosz@biol.uni.lodz.pl (G.B.)

**Keywords:** high resolution melting, HRM limitations, gene scanning, genotyping

## Abstract

High resolution melting (HRM) is a convenient method for gene scanning as well as genotyping of individual and multiple single nucleotide polymorphisms (SNPs). This rapid, simple, closed-tube, homogenous, and cost-efficient approach has the capacity for high specificity and sensitivity, while allowing easy transition to high-throughput scale. In this paper, we provide examples from our laboratory practice of some problematic issues which can affect the performance and data analysis of HRM results, especially with regard to reference curve-based targeted genotyping. We present those examples in order of the typical experimental workflow, and discuss the crucial significance of the respective experimental errors and limitations for the quality and analysis of results. The experimental details which have a decisive impact on correct execution of a HRM genotyping experiment include type and quality of DNA source material, reproducibility of isolation method and template DNA preparation, primer and amplicon design, automation-derived preparation and pipetting inconsistencies, as well as physical limitations in melting curve distinction for alternative variants and careful selection of samples for validation by sequencing. We provide a case-by-case analysis and discussion of actual problems we encountered and solutions that should be taken into account by researchers newly attempting HRM genotyping, especially in a high-throughput setup.

## 1. Introduction

High resolution melting (HRM) analysis generates DNA melt curve profiles which are both specific and sensitive enough to distinguish nucleic acid variation in an exploratory setting (mutation scanning—discovery of unknown genetic variation) and in routine detection of known variants (targeted genotyping based on probes or the more unbiased technique of reference curve-based genotyping of defined multiple single nucleotide polymorphisms (SNPs)) [1]. The multiple benefits of this method have resulted in its many applications in genetic analysis, including microbiological applications [2], such as yeast identification [3,4], mycobacterial species differentiation [5,6], the rapid identification of other bacterial species [7,8] and strains [9], and it is also used in plant genetic research [10] or even food analysis [11]. However, the main interest is focused on human genome analysis and the search for mutations involved in genetic disorders and cancer susceptibility, as well as screening other genes which are clinically interesting [1,12,13]. Such wide application of this method clearly proves its advantages and potential.

HRM analysis has some limitations, like all genotyping methods [14]. Many of them have previously been reviewed or verified in individual studies focusing on selected problems. Much of the work in our laboratory is based on HRM genotyping and gene scanning, and we have encountered, in practice, a number of important limitations and caveats, which can affect the quality of results or impede data analysis using this method. We consider it expedient to present a consistent, structured account of some of these issues for the benefit of researchers initiating HRM experiments or expanding the number of samples, since not all of these limitations were taken into consideration in a systematic manner in previous papers focusing on this topic. In this study, we demonstrate on actual experimental results, the practical consequences of some limitations that have previously been addressed mostly theoretically, which should be of interest for any laboratory performing HRM analysis.

## 2. Results

### 2.1. DNA Source Issues—Problems with Formalin-Fixed, Paraffin-Embedded (FFPE)-Derived Template DNA

**Figure 1:**
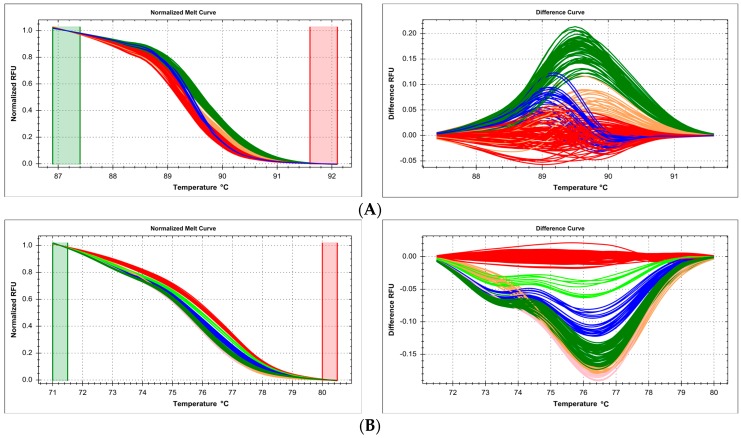

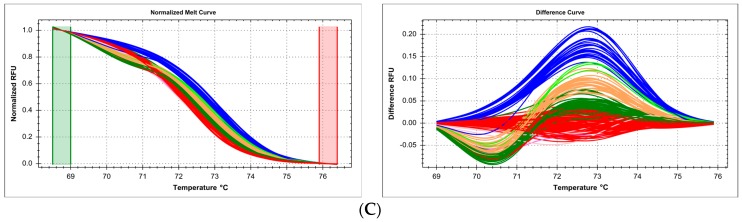
Summary of genotyping of three single nucleotide polymorhisms by high resolution melting (HRM) in samples isolated from formalin-fixed, paraffin-embedded (FFPE) tissue, presented on normalized and difference melting curves plots, fluorescence expressed in relative fluoresce units (RFU). (**A**) c.722C>T (p.Thr241Met, rs861539) in *XRCC3* gene; (**B**) c.2612C>A/G/T (p.Pro871Arg/Gln/Leu, rs799917) in *BRCA1* gene; (**C**) c.3113A>G (p.Glu1038Gly, rs16941) in *BRCA1* gene. Clusters were differentiated with poor resolution and partially overlapped each other.

While DNA from challenging sources (ancient DNA, old clinical samples, nucleic acids damaged by chemicals or radiation) has been successfully used for genotyping by various methods, high-throughput HRM seems to be susceptible to corresponding variations in DNA quality in parallel samples, leading to inconsistent discrimination in melt curve analysis and unreliable genotyping. In our case, DNA obtained from 190 formalin-fixed, paraffin-embedded (FFPE) tissue samples was used in independent genotyping reactions of several SNPs: c.722C>T (p.Thr241Met, rs861539) in *XRCC3* gene, c.2612C>A/G/T (p.Pro871Arg/Gln/Leu, rs799917) and c.3113A>G (p.Glu1038Gly, rs16941) in *BRCA1* gene. Clustering using DNA samples from this material was very poor, the reference curve clusters partially overlapped the polymorphic sample clusters, and thus, the determination of the exact number of variants and unequivocal assignment of samples was impossible (Figure 1). All of these genotyping methods (reaction setups) were designed previously using good quality DNA (isolated from saliva), and worked properly.

### 2.2. DNA Preparation Issues—Different Isolation Methods and Sample Composition Make Parallel Analysis Difficult

**Figure 2:**
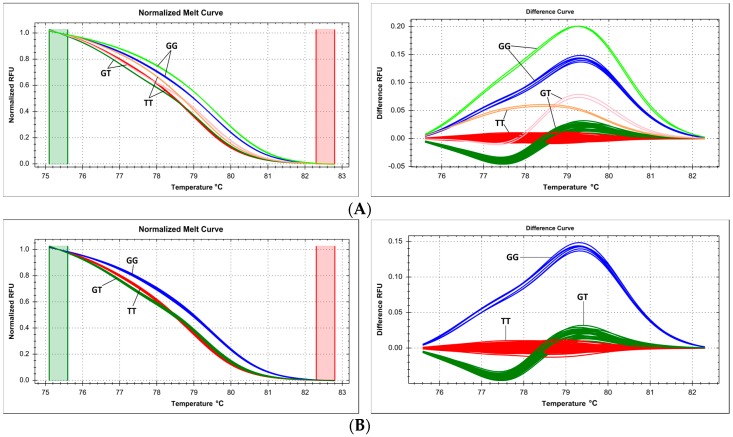

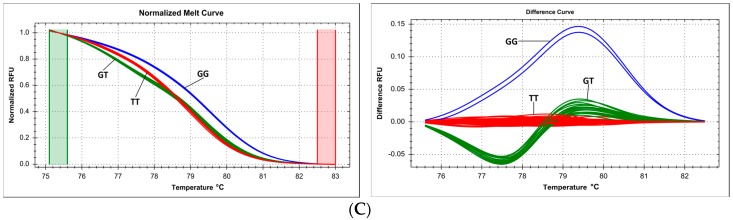
Summary of genotyping SNP g.638144G>T (rs4854344) in *TMEM18* gene by HRM, presented on normalized and difference melting curve plots, fluorescence expressed in relative fluoresce units (RFU). (**A**) Six melting curve clusters derived from two sets of samples from two different DNA isolation methods analyzed together, each genotype is represented by two clusters; (**B**) three melting curve clusters for samples from automatic DNA isolation method on Roche MagNA Pure LC; (**C**) three melting curve clusters for samples from manual DNA isolation method.

DNA samples obtained using various isolation methods can theoretically be used in the same genotyping reaction and analyzed in parallel. We used HRM analysis to genotype the transmembrane protein 18 (*TMEM18*) gene SNP rs4854344 (g.638144G>T), following the protocol of Rosset, et al. [15]. We analyzed in a common workflow (on a single multiwell plate) DNA samples isolated in an automated instrument using magnetic beads (suspended in supplier's elution buffer) and samples manually isolated using isopropanol–ethanol purification (suspended in pure water). Melt curve analysis distinguished six separate clusters, three each (two homozygotes and heterozygote) with samples from each DNA isolation method (Figure 2).

**Figure 3:**
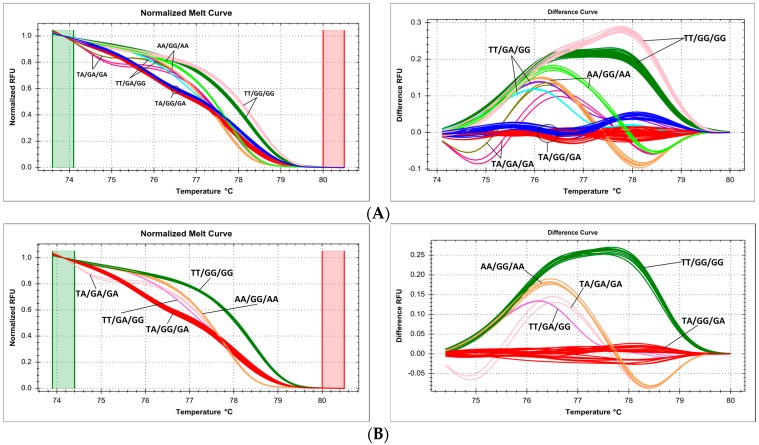

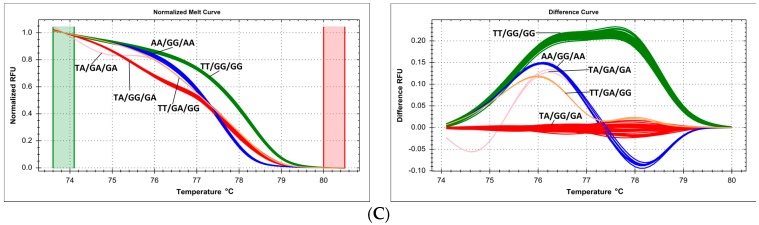
Summary of genotyping of three SNPs in *FTO* gene, c.46-23525T>A (rs9939609), c.46-23540G>A (rs76804286) and c.46-23549G>A (rs9926289), simultaneously by HRM, presented on normalized and difference melting curve plots, fluorescence expressed in relative fluoresce units (RFU). (**A**) Ten melting curve clusters derived from two sets of samples from two different DNA isolation methods analyzed together, each genotype is represented by 2 clusters; (**B**) five melting curve clusters for samples from automatic DNA isolation method on Roche MagNA Pure LC (Roche, Basel, Switzerland); (**C**) five melting curve clusters for samples from manual DNA isolation method.

In a more complicated setting, we replicated the same defect in cluster analysis: genotyping of three separate SNPs of the *FTO* gene, c.46-23525T>A (rs9939609), c.46-23540G>A (rs76804286), and c.46-23549G>A (rs9926289), following the method of Sitek et al. [16], reveals ten different clusters during HRM curve analysis (including some overlap between individual clusters), five for each of the DNA isolation methods (Figure 3). Each set of genotypes (TT/GG/GG, AA/GG/AA, TA/GG/GA, TA/GA/GA, TT/GA/GG) was represented as two separate clusters corresponding to the different DNA isolation methods, while selecting only samples from the same isolation method for analysis guaranteed a clean and unequivocal sample clustering.

### 2.3. Primer Design Issues—Shorter Amplicon Length Is Better

**Figure 4:**
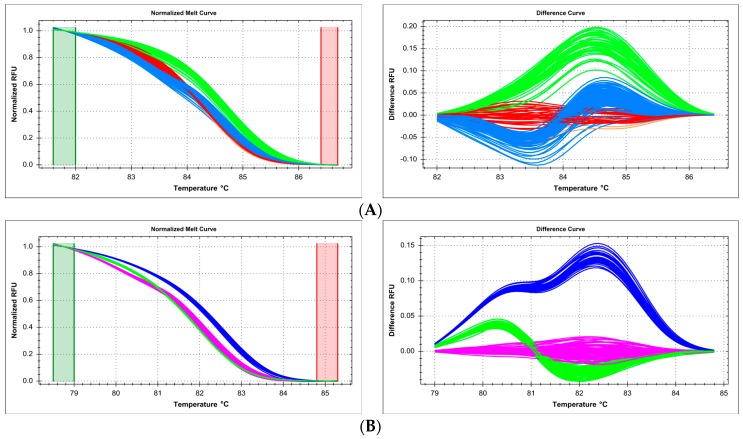
Summary of genotyping of SNP c.3435C>T (p.Ile1145=, rs1045642) in *ABCB1* gene by HRM, presented on normalized and difference melting curve plots, fluorescence expressed in relative fluoresce units (RFU). (**A**) Length of amplicon 108 bp, poor resolution of melting curves; (**B**) length of amplicon 74 bp, much better resolution of melting curves, blue—CC, pink—CT, green—TT.

The expected impact of amplicon length on melting curve resolution was corroborated during targeted genotyping of the SNP c.3435C>T (p.Ile1145=, rs1045642) in the *ABCB1* gene. Initially designed primers with amplicon length of 108 bp lead to weak clustering resolution and partial melt curve cluster overlap (Figure 4A). The primers were redesigned with a reduction of amplicon length to 74 bp, which significantly improved clustering, allowing us to assign all samples into three well-defined clusters corresponding to each genotype (CC/CT/TT, Figure 4B).

### 2.4. Primer Design Issues—Unexpected Variation within Primer Sequence

**Figure 5:**
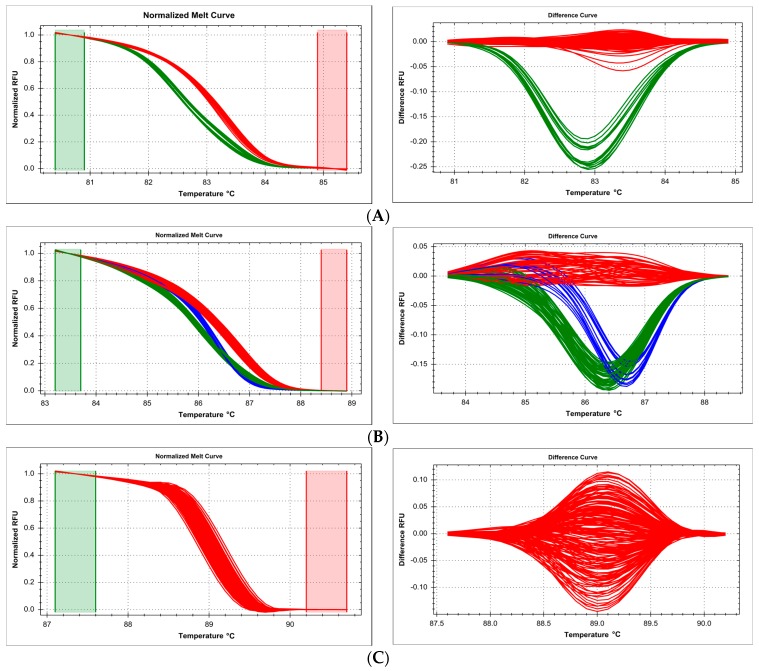

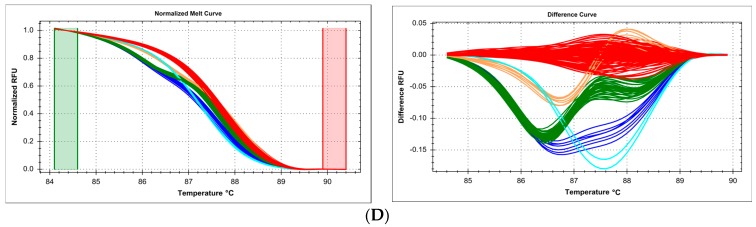
Summary of gene scanning in *ABCC1* gene where variants occurred within sequence complementary to used primers, presented on normalized and difference melting curve plots, fluorescence expressed in relative fluoresce units (RFU). (**A**) Initial scanning of a part of the *ABCC1* exon 4: the differentiated clusters did not correspond to any variation within the scanned sequence between primers; (**B**) genotyping this locus with redesigned primers correctly differentiated SNP c.352-66T>C (rs4148337) within the original forward primer: blue—TT, green—TC, red—CC; (**C**) initial scanning of a part of the *ABCC1* exon 19: no melting curve differentiation; (**D**) genotyping this locus with redesigned primers correctly differentiated a number of SNPs: red—c.2461-30C>G (rs2074087) homozygote, green—c.2461-30C>G heterozygote, blue—c.2461-30C>G homozygote and c.2461-27G>A (rs45492500) heterozygote, cyan—c.2461-30C>G homozygote and c.2461-27G>A homozygote, orange—c.2461-30C>G homozygote and c.2461-39_2461-38delAT (rs45607032) heterozygote.

When designing a targeted genotyping or gene scanning experiment, the location and sequence of primers around the targeted sequence can be arbitrarily selected (with some constraints related to PCR conditions). However, there is a risk of genetic variation within the sequence complementary to the primer, which can lead to incorrect clustering or variant assignment during HRM analysis. In our case, exploratory scanning of a part of exon 4 in the *ABCC1* gene in the Polish population revealed two ostensible melting clusters (containing 13 and 176 samples, respectively; Figure 5A). However, sequencing representative samples showed that there was no actual variation within the scanned sequence area (between primers). On the other hand, three variants of the unexpected intronic SNP c.352-66T>C (rs4148337) have been detected upon sequencing, occurring within the sequence complementary to the forward primer. The original HRM cluster analysis was unable to distinguish these variants, with the two clusters containing a mix of different genotypes. For this reason, we genotyped this locus by redesigning another set of primers around the variation site and successfully performing HRM analysis with well-differentiated curves, obtaining the following genotype distribution: TT—15, TC—80, CC—91 (Figure 5B).

A similar case occurred during scanning of the exon 19 in the same gene, when all samples had initially been classified as one cluster. After sequencing, it turned out that some of them harbored various intronic SNPs located in the sequence complementary to the forward primer (again, no variation has been detected within the scanned area). Redesigned HRM targeted to this polymorphic region yielded good cluster discrimination, and identified c.2461-27G>A (rs45492500), c.2461-30C>G (rs2074087), and c.2461-39_2461-38delAT (rs45607032), with a complicated genotype distribution (Figure 5C,D).

### 2.5. Technical Issues with Experimental Mixture Preparation—Master Mix Reproducibility and Systematic Errors of Automated Pipetting

**Figure 6:**
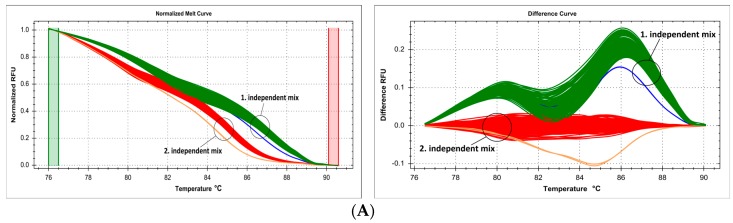

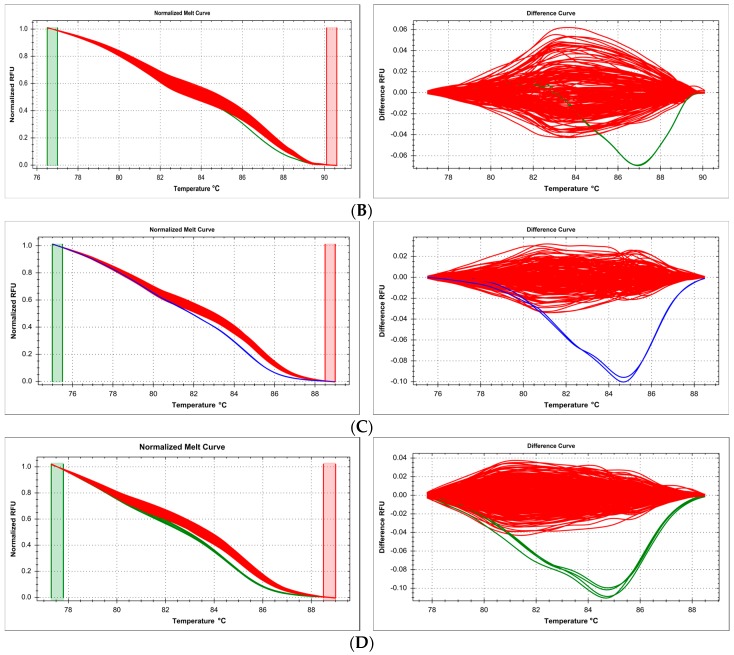
Summary of scanning of *RORC* gene promoter region by HRM, presented on normalized and difference melting curve plots, fluorescence expressed in relative fluoresce units (RFU). (**A**) Four melting clusters of two sets of samples, 190 in total, reaction mixture was prepared for each set independently; clusters red and green—no variants; clusters orange and blue—heterozygous variant c.7+25G>T (rs116171003); (**B**) two melting clusters for set of 95 samples prepared by first independent reaction mixture; cluster red—no variants; cluster green—heterozygous variant c.7+25G>T (rs116171003); (**C**) two melting clusters for set of 95 samples prepared by second independent reaction mixture; cluster red—no variants; cluster blue—heterozygous variant c.7+25G>T (rs116171003); (**D**) two melting clusters of two sets of samples, 190 in total, common reaction mixture was prepared for both sets; cluster red—no variants; cluster green—heterozygous variant c.7+25G>T (rs116171003).

Sometimes, samples are combined into a large experimental run even though they have been prepared on separate occasions, using separately (even if identically) prepared reagent master mixes. In our case, targeted genotyping in the *RORC* gene was conducted for two sets of 96 samples, following the method of Ratajewski, et al. [17]. PCR-HRM reaction was performed on one 384-well HRM plate, but for each of the sample sets, the reaction mixture was prepared independently (according to the same protocol). Even though the samples corresponded only to two genotypes of the SNP c.7+25G>T (rs116171003), melt curve cluster analysis yielded four clearly defined clusters, corresponding to genotypes GG and GT from each sample set/reaction mix (GG—93 and GT—1 in one set, GG—94 and GT—1 in the other set, Figure 6A). Separate analysis of each set was unequivocal and correct (Figure 6B,C). This spurious distinction disappeared completely when only one reaction mixture was used for all samples, and HRM was performed on a whole 384-well plate, leading to correct identification of two clusters (GG—187 and GT—2, Figure 6D).

**Figure 7:**
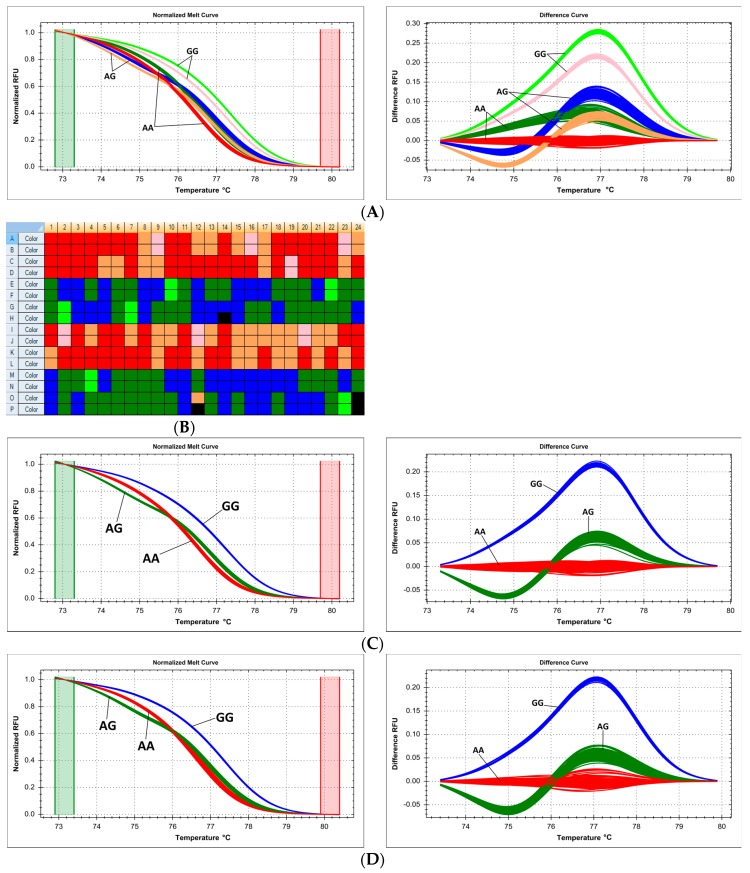
Summary of genotyping of the SNP c.1016+213A>G (rs6684205) in *TGFB2* gene by HRM, presented on normalized and difference melting curve plots, fluorescence expressed in relative fluorescence units (RFU). (**A**) 6 melting clusters differentiated from the sample set, using all eight channels of the pipetting robot; (**B**) plate view of HRM genotyping clusters, colors correspond to curves in Figure 7A; pipetting head duplicates samples vertically: channel 1 pipets into rows A, B, channel 2—C, D, channel 3—E, F, channel 4—G, H, channel 5—I, J, channel 6—K, L, channel 7—M, N, channel 8—O, P; (**C**) three melting clusters for the samples prepared by robotic channels 1, 2, 5, 6; (**D**) three melting clusters for the robotic channels 3, 4, 7, 8.

When performing a large high-throughput genotyping experiment, pipetting of reagents and samples is often left to automated equipment. However, due to sensitivity of the HRM method, even small systematic errors can lead to problems in melt curve clustering and assignment. In our case, we used a robotic pipetting system which was regularly validated, and its pipetting variability was found to be within the permissible range. Still, during targeted HRM genotyping of the SNP c.1016+213A>G (rs6684205) in *TGFB2* gene, instead of expected three clusters, six independent clusters were generated (Figure 7A). We determined that three of the clusters were obtained for samples prepared by pipetting channels/heads 1, 2, 5, 6, while the remaining three clusters corresponded to channels/heads 3, 4, 7, 8 (Figure 7B). Each of the three detected genotypes (AA/AG/GG) was represented by two distinct clusters, one for each set of robot channels (Figure 7C,D). Interestingly, this effect was not observed on other targeted genotyping reactions run on the same automated equipment in the same period of time, reflecting the varying sensitivity of various HRM reactions.

### 2.6. Issues with Multiple Proximate Polymorphic Sites—Complex Melting Curves Lead to Underestimation of the Prevalence of Variants within a Single Amplicon

**Figure 8:**
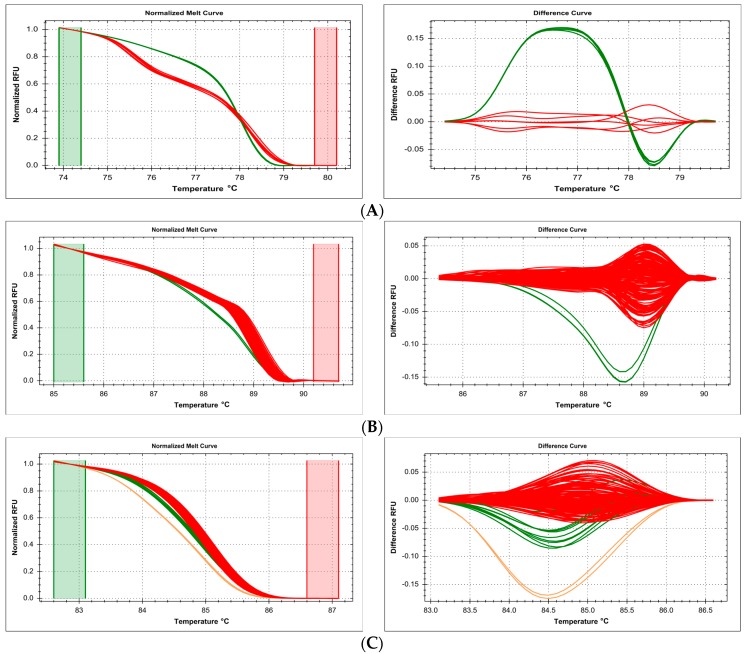
Summary of scanning genes’ fragments in which different variants, of the same type, formed one melting cluster. Results presented on normalized and difference melting curve plots, fluorescence expressed in relative fluoresce units (RFU). (**A**) Scanning of a part of *Candida albicans ERG11* gene for 315T>C and 411C>T polymorphisms, green—undistinguishable reference homozygote (TT/CC) and double variant homozygote (CC/TT), red—heterozygous samples; (**B**) scanning of the human *ABCC1* exon 27 for c.3886C>T (p.Arg1296Trp, rs200922662) and c.3901C>T (p.Arg1301Cys, rs201533167) polymorphisms, red—reference homozygote (CC/CC), green—two different undistinguishable heterozygotes (CT/CC and CC/CT), (**C**) scanning of the *ABCG2* exon 11 for c.1278-28G>A (rs771435451), c.1302G>A (p.Thr434=, rs781367109) and c.1367+20G>A (rs2231153), red—reference homozygote (GG/GG/GG), green—undistinguishable heterozygotes GA/GG/GG and GG/GG/GA, pink—heterozygote GG/GA/GG.

An inherent feature of the HRM method, where the shape of the melting curve depends on nucleotide composition of the amplicon, is the fact that proximate mutations, with reciprocally compensatory effect with regard to nucleotide composition, may cancel each other out with regard to melting curve shape. During scanning of one area the *Candida albicans ERG11* gene, two separate clusters were distinguished (Figure 8A). Unexpectedly, it turned out that one of them contained samples with two different genetic variants: double homozygotes for proximate reciprocal (315T>C/411C>T) polymorphisms within the same amplicon. The effect of nucleotide neighborhood on melting curve shape was insufficient to distinguish the TT/CC from the CC/TT genotype. The other melting curve cluster contained heterozygotic samples.

Scanning of the *ABCC1* exon 27 in the Polish population revealed two separate HRM curve clusters. One of them included samples which turned out to represent two different heterozygous variants, caused by the same type of nucleotide alteration (C>T): c.3901C>T (p.Arg1301Cys, rs201533167) and c.3886C>T (p.Arg1296Trp, rs200922662) (Figure 8B). The other cluster included double homozygotes of reference (wild type) variants.

Another complex example of obscured polymorphisms occurred during scanning of the *ABCG2* exon 11 in the Polish population. In this scanned area, three proximate heterozygous polymorphisms have been detected, all with the G>A nucleotide change. Interestingly, one of these variants (c.1302G>A, p.Thr434=, rs781367109) changed the shape of the melting curve in a distinct manner, allowing for the differentiation of a separate cluster in a heterozygous sample. However, the other two variants c.1278-28G>A (rs771435451) and c.1367+20G>A (rs2231153) in their heterozygous form generated melt curves that were classified to the same cluster (Figure 8C).

## 3. Discussion

Since the introduction of HRM method into common laboratory use, which was initiated by the development of LCGreen dye (BioFire Diagnostics, Inc., Salt Lake, UT, USA) [18], there has been a rapid development of intercalating dyes, equipment and software to perform HRM analysis, and apply it for genotyping. Currently, several different platforms are available, e.g., LightScanner System (BioFire Diagnostics, Inc., Salt Lake, UT, USA), Roche LightCycler System, Bio-Rad CFX™ Real-Time PCR Detection System, Rotor-Gene G (Qiagen, Hilden, Germany). While HRM fundamentals are common to all these instruments, specifics of experimental setup, and above all, data analysis, are divergent, and require separate approaches, especially with regard to optimization. Systems and reagents have been compared in other studies, and we do not aim here to argue for the superiority of any of the systems [19,20,21,22,23,24]. Selection of the most suitable system and reagents depends strongly on the type of genetic analysis to be performed, the required capacity, and other details of the scientific problem that is to be solved [25].

In the present study, we decided to focus on pitfalls and caveats that await a molecular geneticist who intends to use the HRM method to study genetic variation, especially in a large-scale (high-throughput) setting. There are three main approaches to researching sequence variants by HRM: gene scanning (a mainly exploratory method that concentrates on finding hitherto unknown variants), probe-based directed genotyping (where the presence or absence of a specific mutation can be directly determined on the basis of designed probe sequence) and reference curve-based directed genotyping, where one of possible genotypes is selected as reference (“wild type”), and specific deviations from it are subsequently identified and assigned to alternative alleles/genotypes. HRM is an especially valuable method for the latter approach, where it has few alternatives with comparable power, throughput, and economic value [26,27]. Therefore, we put together examples from our own work using this type of workflow, and presented them in a comprehensive logical listing of practical caveats and limitations which are sometimes overlooked by those with limited experience with this particular method. We aim to present and discuss examples of failures or complications in data generation and/or analysis in HRM-based genotyping, and reiterate in a systematic manner, some rules that have to be followed since they can have a crucial impact on the quality of results and their analysis [28]. In Table 1, we also summarized all of this troubleshooting combined as a solution, with helpful guidance for every scientist who would be interested in using HRM technique.

It has been stressed before that the source of template DNA and the way it is prepared is vitally important for the success of any genotyping experiment [29,30]. We provide practical examples that are especially important in large-scale directed genotyping by HRM curve fitting and analysis, where reproducibility is key to correct mathematical fitting and clustering of curves, and thus, to reliable genotype assignment. We demonstrate the type of results that are obtained from poor quality DNA isolated from FFPE tissue samples (Figure 1): with a small number of samples, fitting algorithms are statistically likely to deal properly with the task of distinguishing genotypes, but upon scale-up, the variability of readout leads to inadmissible noise-to-signal ratios and overlapping clustering criteria, which may lead to erroneous genotype assignment. Even ascertaining the number of distinct genotypes was sometimes difficult, though the experiments involved targeted genotyping of a specific polymorphism, and not introductory gene scanning. Of course, this does not mean that FFPE-derived DNA is entirely unsuitable for HRM-based genotyping—however, a less naive approach must be taken, as demonstrated by others who successfully applied e.g., the sophisticated strategy of HRM-SNaPshot [31]. When the number of samples and the number of possible genotypes is smaller, and when the samples have been both preserved and subsequently isolated in a reproducible manner, preferably by the same person in a single batch, the chance of success in HRM-based genotyping is much higher, as seen e.g., in the studies on specific SNPs related with cancer susceptibility [32,33,34].

The problem of DNA template reproducibility extends to the standardization of sample form before the closed-tube PCR and HRM phases of the method [35]. DNA concentration must be standardized for all samples, and DNA has to be dissolved in the same solvent for all simultaneously analyzed samples, which is usually a direct function and consequence of the applied isolation method [36]. We compared, on the same microplate in parallel, HRM profiles of samples derived from two DNA isolation methods, and consequently dissolved in different solvents (Figure 2 and Figure 3). Even though the dilution factor of the DNA sample in the PCR mix was very high, and thus, the impact of solvent matrix was thought to be negligible, we show that each of these samples sets gave slightly different melting curves and required separate analysis. While this problem has been raised before, e.g., with regard to the fact that DNA obtained by some isolation methods can give false positive results during analysis [29], we concur with other experts in recommending that more attention is to be paid to this question in routine analysis of a large number of samples [37,38].

Another question that needs to be approached even before starting the experiment itself is primer design. Though rules for PCR-HRM primer design are well-known and widely available [39], some specific consequences of giving yourself too much leeway (or just bad luck), in this respect, may not be obvious. Amplicon length is the most discussed HRM limitation. PCR products up to 300–400 bp can melt with enough sensitivity and specificity, generating up to 100% correct assignment, but for longer products these parameters decreased (especially sensitivity) [40]. However, for routine work, even shorter fragments are recommended, 150–250 bp for gene scanning and 80–100 bp for targeted genotyping [25]. From the point of view of DNA chemistry, this is critical for high sensitivity because such fragments usually contain one melt domain. When applying HRM to longer fragments that contain several melt domains, discriminative power of curve fitting, and thus, the chance of distinguishing variants decreases [41]. Analysis of amplicons over 500 bp is very difficult, because they often present gradual melting, disrupting variant detection. For this reason, genotyping fragments containing multiple melt domains or scanning long exons should be avoided, and they should be divided into shorter amplicons [1]. Reduction of amplicon size leads to increase of relative melting temperature differences, and thereby, better differentiation [12]. In a previous study, we compared scanning and genotyping HRM results for ten *ABCC1* SNPs, to validate the accuracy of both approaches. We were able to show that we obtain reliable genotype assignments irrespective of size difference of PCR amplicons, which were 41–125 bp for targeted genotyping of SNPs and 99–248 bp for exon scanning [42]. However, in the present study, we present an example of targeted genotyping of an SNP (rs1045642 in *ABCB1*), where the original PCR product, 108 bp long, melted with poor curve resolution, and reduction of amplicon size allowed for a more accurate analysis (Figure 4). This shows that it is difficult to recommend a rigid limit of safe amplicon length, and it should be optimized based on initially obtained results, as in our example. As a rule, primers used for targeted genotyping should be designed as close to the SNP as possible, shortening the amplicon and simplifying the assay.

We also present examples of melt curve clustering problems and inconsistencies which arise when unplanned SNPs are present in the sequence complementary to the primer (Figure 5). Samples with such variants were not completely differentiated or incorrectly clustered. It must be recognized that the HRM method, in principle, does not allow for correct detection of polymorphisms in sequence complementary to the primers used. When this situation arises, primers should be redesigned, or additional primer pairs which flank the SNP position should be included in experimental setup. Our results demonstrate the importance of direct validation of dubious results by sequencing, not only targeted at the SNP which is planned for genotyping, or at the “scanned area” between the primers, but at the entire amplicon length, to safeguard against such pitfalls.

One of the underreported technical issues which affect results of HRM-based genotype analysis is reproducibility of pipetting. While volumetric consistency is important for any kind of molecular biology experiment, and glaring errors in that respect may compromise any genotyping method, the closed-tube character of PCR-HRM and large sensitivity of melt curve shape to very small changes in environmental factors (including, but not limited to, pH, ionic force, cation concentration, etc.) make otherwise routine pipetting variability inadmissible in this method. We present two examples: separate reagent master mix preparation (a routine occurrence in large-scale experiment which is not expected to impact results qualitatively or quantitatively) and preparation of PCR mix by automated (robotic) pipetting equipment (Figure 6 and Figure 7). In both cases, small pipetting divergences which were deemed to be within admissible variability ranges for other methods led to significant difficulties in correct genotype assignment (HRM curve fitting and clustering). Tucker and Huynh [39] similarly reported that accurate pipetting in all wells to ensure an equal volume and the same concentration of reaction components is indispensable, because differences, in particular in salt concentration, can result in alterations in DNA melting behavior. Therefore, we recommend preparing one large-volume reagent master mix for all samples in a single experiment, and especially, strict volumetric quality control of all manual and automated pipetting equipment when performing large-scale genotyping by the HRM method. Duplicating samples in various channels of automated pipettors should be a routine procedure. This is very important, and should be taken into account by newcomers to the technology, since there are literature reports which conclude that reaction volume (mixture + template) or template concentration does not impact melting reproducibility; however, they have not verified the effect of independently preparing partial reagent mixtures or multi-channel automated pipetting [43,44,45].

Another pitfall that might not be immediately obvious is related to variants that cannot be distinguished using the HRM method. It is known that some nucleotide changes can give highly similar profiles, especially A>T/T>A, where subtle differences may become indistinguishable on some instruments [25]. In this situation, sample mixing to generate hetero-duplexes is recommended, as this increases the recorded difference [25]. In this study, we present two different types of traps that lead to erroneous false negative (not detecting an existing variability) results (Figure 8). In one case, a double homozygotic variant with regard to two reciprocal mutations (T>C in one locus, C>T in the other one) was indistinguishable from the reference curve—since melting characteristics are influenced more strongly by nucleotide composition than by sequence neighborhood [46], this behavior was statistically probable, and its actual practical occurrence is not surprising. In other cases, we show that two identical (G>A) variants within the same amplicon were indistinguishable from each other when heterozygotic—however, interestingly, a third identical variant in the same amplicon could be distinguished. There is evidence that variant location in the amplicon is sometimes significant—in our case, the more sensitively detected variant is close to the center of the amplicon. This is consistent with the conclusion of other authors who observed the greatest variant impact in central amplicon location [46]. However, there is other ample data showing that the location of the mutation in the amplicon is not relevant [40,47,48], and we have numerous examples of this more expected behavior in our work as well. Therefore, it is important to validate your method carefully, before embarking on a high-throughput application: direct sequencing plays a crucial role for HRM result verification, and should be used as a golden standard. While it is recognized that each unknown sample representing a unique melting curve should be verified by sequencing [1], our results show that sometimes, samples with variants which are masked by another variant can co-cluster with the reference curve or with each other—other similar examples can be found in the literature [49]. For cost-related and practical reasons, full sequencing is only applicable to clusters with few samples [50]—however, testing a statistically significant selection of samples, whenever a possible masking variant is suspected, should become routine procedure. For the same reasons, direct sequencing is advisable only for a small series (up to 10 samples), but for larger ones, HRM becomes more and more cost-efficient. However, it is important to remember that in complex clinical samples, when the level of variant-containing DNA in the sample is lower than 50% (e.g., in leukemia diagnostics), the sensitivity of direct sequencing decreases dramatically, and can be even lower than HRM, so in these cases, sequencing cannot be considered as a gold standard for validation [51].

In conclusion, HRM is a rapid, simple, and cost-efficient method for high-throughput genotyping, applicable both to mutation scanning and large-scale population analysis targeted to individual SNPs. HRM demonstrated its high specificity and sensitivity in practical use, both in basic research and in clinical diagnostics, and it is still much cheaper than more comprehensive molecular technologies like NGS. As a closed-tube homogenous system, it ensures that the whole reaction and analysis is done without additional processing or separation steps, which protects against contamination—a scourge of many other methods. However, when performing a HRM genotyping experiment, one should keep in mind some limitations and caveats, which may not be obvious at first glance or adequately addressed in a systematic manner in available documentation. Therefore, we present examples of potential pitfalls and compromised results for the consideration of researchers which are either new to the technique or are planning to significantly increase the number of processed samples.

## 4. Materials and Methods

### 4.1. Materials and Genomic DNA Samples

Human genomic DNA samples were derived from anonymous Polish unrelated volunteers. Samples were randomly selected for each experiment from a 10,000-strong cohort of the “normal Polish population” held in genetic collection at the Biobank Lab, Department of Molecular Biophysics, University of Lodz. Genetic material for this collection was sampled in 2011–2012 within the EU-funded TESTOPLEK project. This collection was involved in creation of a retrospective POPULOUS collection (POPUlation—LOdz UniverSity Biobank), and registered since 2013 in the BBMRI catalog of population collections [52]. All subjects gave their written informed consent to participate in the study. This study was approved by the regional ethical committee (Institutional Review Board of the University of Łódź) and all procedures were performed in accordance with the Declaration of Helsinki.

Saliva was collected into Oragene OG-500 DNA collection/storage receptacles (DNA Genotek, Kanata, ON, Canada), and genomic DNA was subsequently isolated by magnetic beads using the MagNA Pure LC DNA Isolation Kit—Large Volume (Roche, Basel, Switzerland) with final concentration normalized to 200 pg/µL, suspended in elution buffer (supplied in kit). Another method of DNA isolation from saliva was adopted for some of the samples, using the manufacturers protocol for manual isopropanol–ethanol purification on a deep 96-well plate [53]. These samples were also normalized to 200 pg/µL in pure water.

DNA isolation from FFPE tissue samples was performed according to the following protocol. Xylene (800 µL) was added to FFPE tissue samples. They were incubated for 10 min at room temperature with gentle mixing and centrifuged for 10 min (13,200 rpm). Supernatant was removed. This procedure was repeated once more. Subsequently, 400 µL xylene and 400 µL ethanol (99.8%) were added to the samples. They were incubated for 5 min at room temperature with gentle mixing and centrifuged for 5 min (13,200 rpm). Supernatant was removed. Ethanol (800 µL; 99.8%) was added to the samples. They were incubated for 5 min at room temperature with gentle mixing and centrifuged for 5 min (13,200 rpm). Supernatant was removed. Ethanol (800 µL; 70%) was added to the samples. They were incubated for 5 min at room temperature with gentle mixing and centrifuged for 5 min (13,200 rpm). Supernatant was removed. Ethanol (800 µL; 50%) was added to the samples. Samples were incubated for 5 min at room temperature with gentle mixing and centrifuged for 5 min (13,200 rpm). Supernatant was removed. Pellets were dried in thermomixer for 15 min at 55 °C, suspended in TE buffer, and DNA concentration was normalized to 200 pg/µL.

*Candida albicans* DNA was obtained according to the method described previously by Caban et al. [54].

### 4.2. Detection of Polymorphisms Using High Resolution Melting (HRM) Scanning and Genotyping

For each of the polymorphisms described in this study, for different genes, the following parameters obtained from GenBank [55] were assigned: dbSNP IDs (rs numbers), coding DNA nucleotide position within the NM reference sequence, amino acid position in the protein for SNPs in exons within the NP reference sequence, genomic nucleotide position within the NC reference sequence for polymorphisms in non-coding DNA, and for fungal DNA, nucleotide position within the NW annotated genomic reference sequence. The respective reference sequences are listed below: ABC transporter gene *ABCC1* (or multidrug resistance-associated protein 1—MRP1, NM_004996.3, NP_004987.2), ABC transporter gene *ABCG2* (or breast cancer resistance protein—BCRP, NM_004827.2, NP_004818.2), RAR-related orphan receptor C gene (*RORC*, NM_001001523.1) and *C. albicans* lanosterol 14-α-demethylase gene (*ERG11*, NW_139482.1 (Ca21chr5_C_albicans_SC5314:149706 to 148120)). A genotyping strategy was applied for SNPs in: transmembrane protein 18 gene (*TMEM18*, NC_000002.12), fat mass and obesity associated gene (*FTO*, NM_001080432.2), X-ray repair cross complementing 3 gene (*XRCC3*, NM_001100118.1, NP_001093588.1), breast cancer type 1 susceptibility gene (*BRCA1*, NM_007294.3, NP_009225.1), transforming growth factor beta 2 gene (*TGFB2*, NM_001135599.2), and ABC transporter gene *ABCB1* encoding glycoprotein-P (or multidrug resistance protein 1—MDR1, NM_000927.4, NP_000918.2).

### 4.3. HRM Conditions and Analysis

A single, standard reaction mixture (10 µL) was prepared using the Janus^®^ Automated Workstation (Perkin Elmer Inc., Waltham, MA, USA) and was composed of GoTaq^®^ Colorless Master Mix (2×) (Promega, Madison, WI, USA), LC Green Plus^®^ dye (10×) (BioFire Defense, Inc., Salt Lake, UT, USA), 0.5 µL of 10 µM primers mixture, 3 µL DNA (200 pg/µL), and filled up to the final volume with 0.5 µL of water. Reaction was performed on 384-micro well plate using CFX384™ real-time PCR system (Bio-Rad Laboratories Inc., Hercules, CA, USA) (all samples were duplicated). The reaction conditions were as follows: initial denaturation at 95 °C for 3 min, 50 amplification cycles of denaturation at 95 °C for 30 s and annealing at specific temperature depending on the primers used, for 30 s (the list of all used primers in Table 2. The plate was read after each cycle. Directly afterwards, melting curve was determined, by incubating the plate at 90 °C for 60 s, 40 °C for 60 s and from 65 °C to 95 °C with an increment 0.2 °C for 10 s with plate reading. The obtained data was analyzed using Bio-Rad Precision Melt Analysis Software, version 1.2 (Bio-Rad Laboratories Inc.) [42].

In some cases, genetic variation was verified by direct sequencing method for several selected samples representing each cluster. Preparation of samples for sequencing was conducted according to the protocol described previously [42]. Analysis of sequencing results was performed by CodonCode Aligner software (CodonCode Corporation, Centerville, MA, USA) based on the reference sequences (GenBank) corresponding to each respective gene. Sequencing results of selected samples were compared with respective clusters of HRM melting curves, and genetic variation was verified.

## Figures and Tables

**Table 1:** Practical guidance for high resolution melting (HRM) user with troubleshooting and proposals for solutions.

Issue	Troubleshooting	Solutions
DNA source	poor quality results from DNA isolated from FFPE tissue samples	- adequate storage of tissue samples - subsequent isolation in a reproducible manner, preferably by the same person in a single batch - application of more adequate approach e.g., HRM-SNaPshot strategy - if results quality is still poor, there is to consider performance of the next DNA isolation from FFPE tissue samples using more efficient method
DNA preparation	different isolation methods and sample composition make parallel analysis difficult	- using a standardized highly robust DNA extraction method for all samples - dissolution of DNA in the same buffer solution for all simultaneously analyzed samples - standardization of quantification and dilution of all DNA samples - parallel analysis for samples from the same isolation method guaranteed a clean and unequivocal sample clustering
Primer design	poor melting curve resolution and PCR optimization	- standard rules of primers design (a similar melting temperature for all primers, optimally 55–65 °C; avoid secondary structures like hairpins, homodimers or heterodimers) - for targeted genotyping, primers should be designed as close to the SNP as possible; for scanning, at least 20 bases upstream from the 5′-end of the exon and downstream from the 3′-end of the exon - reduction of amplicon size usually improved clustering (recommended 80–100 bp for targeted genotyping and 150–250 bp for gene scanning, however avoid multiple melting domains in one amplicon) - testing reaction with designed primers: recommended PCR optimization with an annealing temperature gradient - performance of electrophoresis after PCR reveals primers specificity, expected size of amplicon and absence from extraneous products - determination the most efficient and specific primers pair for each tested fragment - if necessary, adjust the magnesium ion concentration Mg^2+^ - if possible, test melting curves differentiation for selected samples with known genotypes
	unexpected variation within primer sequence and incorrect clustering	- verification of melting for representative samples by “golden standard” method like Sanger sequencing - redesign primers or additional primer pairs which flank the SNP position (located in the sequence complementary to the primer) should be included in experimental setup
Technical issues with reproducibility	reagents, equipment, handling	- Hot-Start DNA polymerases improve reaction specificity by eliminating nonspecific products - dsDNA binding dyes are photosensitive and should be protected from light - ensure that all reaction components are adequately mixed and centrifuged before pipetting - white PCR plates are preferable to clear plates - stick well an optically clear seal on plate to avoid evaporation and ensure effective light transmitting - centrifugation plate with PCR-HRM samples before place in a real-time instrument
	reproducibility of manual pipetting	- pipets should be calibrated, for large-scale experiment multichannel pipets are recommended (e.g., on 384-well plate); automated pipettor like Repetman^®^ (Gilson, Inc., Madison, WI, USA) is also a very good solution - in case of manual, multiple pipetting of the same reagent, the most accurate method of pipetting should be used, like reverse pipetting technique, which ensure the same pipetting volume each time, and also avoid bubbling - duplicating samples should be a routine procedure as internal control of pipetting, as well as non-template control - preparation of fresh, one and common reagent master mix for all samples in a single experiment
	reproducibility of automated liquid handling instrument	- automated equipment should be calibrated, and quality of pipetting should be verified before approach - duplicating samples should be a routine procedure as internal control of pipetting, as well as non-template control - during subsequent analysis, clustering for samples pipetted by every channel should be performed and compared
Multiple proximate polymorphic sites	underestimation of the prevalence of variants within a single amplicon	- when it is possible, sample mixing is recommended to generate hetero-duplexes during melting - direct sequencing should be used as a golden standard for HRM result verification - each unknown sample representing a unique melting curve should be verified by sequencing

**Table 2:** Primers used for High Resolution Melting methods, scanning and genotyping, and corresponding primers for direct sequencing. Amplicon size includes primers. Due to genotyping failure of the single nucleotide polymorphisms in samples derived from Formalin-Fixed, Paraffin-Embedded tissue samples (description in the main text), primers for direct sequencing have not been designed. (new)—redesigned primer pair (description in the main text).

HRM					Direct Sequencing of the Area of Interest		
Gene	dbSNP ID	Forward Primer (5′→3′)	Reverse Primer (5′→3′)	Size (bp)	Forward Primer (5′→3′)	Reverse Primer (5′→3′)	Size (bp)
*ABCC1*	rs4148337	AGCCTGGGTGACAAGAGTGA	TGGATCTCAGGATGGCTAGG	189	GTGGTGAAACCCCGTCTTTA	CCTTGGAGCAACACAGACAA	604
	rs4148337 (new)	AAGCTGAGGCAGGAGAATCA	AAGGTAGCAAGCAGCTGAGG	163			
	rs2074087	CTCACACATGTGCACTCACG	TCTGTGCTGGCATAGGTACG	204	CATGTCCCACCTTCAGACCT	CCAGCTTAACTCCGTGCTTC	748
	rs2074087 (new) rs45492500 rs45607032	GCCAAGCTAGGCAGTCTCAC	GGCAAGTAGCTCATGCTGTG	99			
	rs201533167 rs200922662	GGGGAGTCACAGCTTTACCA	GGGAATGGGTGAGGGAAT	248	AGGGGACAGAGGGACACAG	AAATCTGTGGGGCTCATTTG	618
*ABCG2*	rs771435451 rs2231153 rs781367109	TGTGGAAAGAGTTTTGTGGGTA	CTAACCAATAGCCCCTGCTG	229	AGGGCCCATCTTCAAATACC	TTGCTTGCTCTCTCCAACATT	732
*RORC*	rs116171003	GTGAATGGGGCCACCTG	GACGACAGGGTCCAGGCT	45	CTCGGGGGTAGGAGGAGTAG	CCATCTCCCAACAGATCTTGA	602
*ERG11*	Exon	TTAGGGAAAATTATGACGGTTTAT	CTTTCATCAGTAACAAAATAATTCAAA	263	TTTTCTTCATCTTACTTCTTTCTTTCA	TTGACCACCCATAAGAATACCA	1082
*TMEM18*	rs4854344	TGTTTAGATACACACTCTCCACTGT	GATGGCTGTGCTGGAACTG	58	TGCGATGAACTGAGTGTTGC	ACCATTTCTGGAACGTGGAG	638
*FTO*	rs9939609 rs76804286 rs9926289	CATCAGTTATGCATTTAGAATGTCTG	AGAGTAACAGAGACTATCCAAGTGC	95	TGGTTTCAGAGGCTTGTGTG	GCCCAAGGATGGTGTTTCTA	695
*XRCC3*	rs861539	TTCCGCTGTGAATTTGACAG	CTCACCTGGTTGATGCACAG	125	-	-	-
*BRCA1*	rs799917	AAAGCGCCAGTCATTTGC	CTTCTGCATTTCCTGGATTTGA	47	-	-	-
	rs16941	GCCGTAATAACATTAGAGAAAATG	TTAATATTGCTTGAGCTGGC	55	-	-	-
*TGFB2*	rs6684205	AGTGAACCAAGTGTGAAGGGA	TTCAGAGGAATTTTGGGGAA	61	TGGCACCTCCACATATACCA	ACGGCATTCTTCTGCTGTCT	612
*ABCB1*	rs1045642	CCTGTTTGACTGCAGCATTG	AAGGCATGTATGTTGGCCTC	108	TGTTTTCAGCTGCTTGATGG	GTGGGGACCCAGACTCTGTA	604
	rs1045642 (new)	GGGTGGTGTCACAGGAAGAG	AGGCAGTGACTCGATGAAGG	74			
